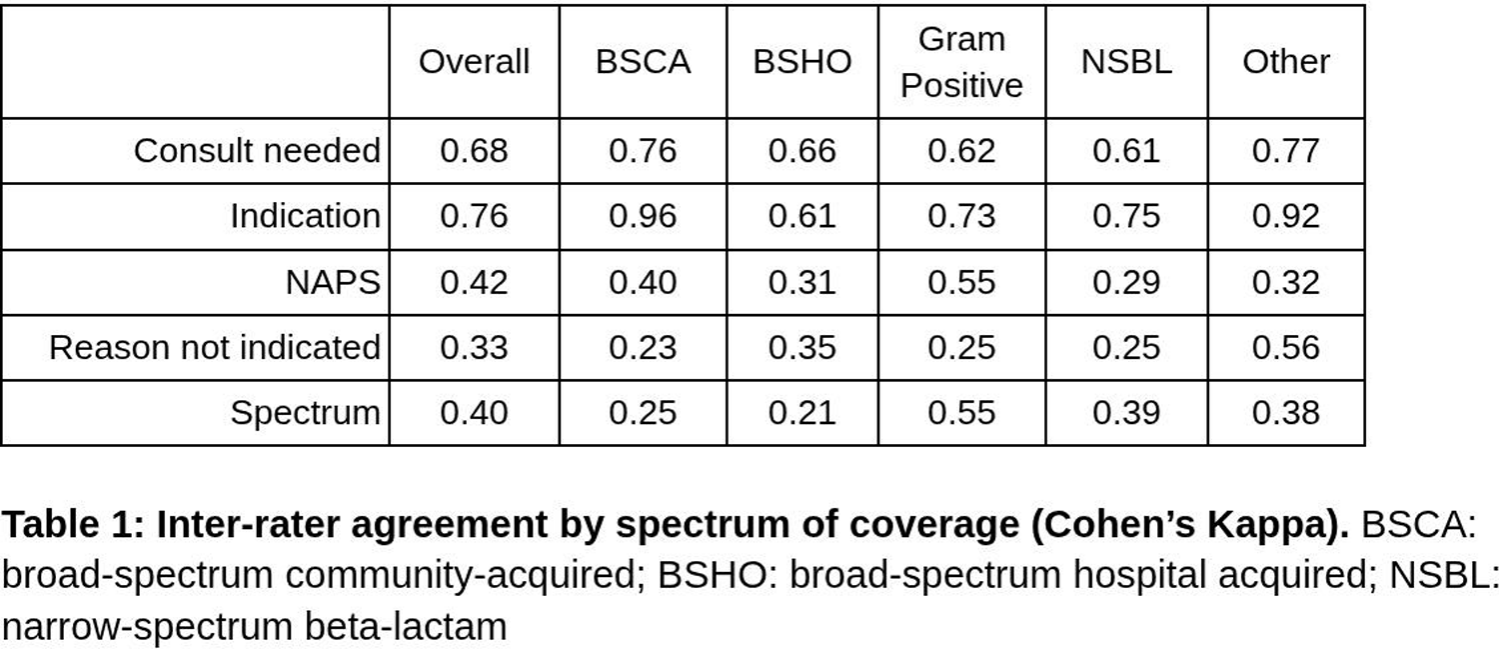# 121 Uncovering Colonization: Multidrug-Resistant Organism Surveillance in a Long-Term Acute Care Hospital, 2024-2025

**DOI:** 10.1017/ash.2026.10535

**Published:** 2026-06-23

**Authors:** Nathan Radakovich, Allison Bond, Natasha Spottiswoode, Ritika Prasad, Daniel Escobar, Emily Kaip, Will Simmons, Sarah Doernberg

**Affiliations:** 1 UCSF; 2 UCSF Health; 3 University of California, San Francisco

## Abstract

**Background:** Prospective audit and feedback (PAF), a cornerstone of antimicrobial stewardship programs (ASP), relies on infectious disease (ID) clinicians’ direct review of electronic health records (EHR). We assessed the consistency of ID clinicians’ EHR-based assessments on real-world patient data. **Methods:** Antibiotic orders were randomly selected from a list of hospitalized adults at UCSF medical center. ID specialists (physicians and pharmacists) independently reviewed orders in real time using data from the EHR. Reviewers assessed the stated need for antibiotics, their agreement with necessity and choice, and overall appropriateness as defined by the National Antimicrobial Prescribing Survey (NAPS) tool. We measured agreement with either Cohen’s kappa or weighted Cohen’s kappa for ordinal observations. **Results:** **Conclusion:** We observed only modest agreement between independent ID clinicians’ reviews of antibiotic orders in a real-world assessment of inter-rater agreement. Discrepancies in assessments likely reflect a mixture of practice pattern variation, the intrinsic difficulty of assessing antibiotic choice without directly evaluating the patient, and variation due to the instruments used to collect information. Reference: Khanina A, Douglas AP, Yeoh DK, et al. Validation of the Antifungal National Antimicrobial Prescribing Survey (AF-NAPS) quality assessment tool. J Antimicrob Chemother. 2023;78(6):1367-1377. doi:10.1093/jac/dkad085

**Figure:**